# Size Does Matter: Why Polyploid Tumor Cells are Critical Drug Targets in the War on Cancer

**DOI:** 10.3389/fonc.2014.00123

**Published:** 2014-05-26

**Authors:** Jermaine Coward, Angus Harding

**Affiliations:** ^1^Mater Medical Research Institute, Princess Alexandra Hospital, Woolloongabba, QLD, Australia; ^2^The University of Queensland Diamantina Institute, The University of Queensland, Translational Research Institute, Brisbane, QLD, Australia

**Keywords:** polyploidy, hyperdiploidy, tumor evolution, therapy resistance, tumor initiation, cancer stem cell, aneuploidy, chromosomal instability

## Abstract

Tumor evolution presents a formidable obstacle that currently prevents the development of truly curative treatments for cancer. In this perspective, we advocate for the hypothesis that tumor cells with significantly elevated genomic content (polyploid tumor cells) facilitate rapid tumor evolution and the acquisition of therapy resistance in multiple incurable cancers. We appeal to studies conducted in yeast, cancer models, and cancer patients, which all converge on the hypothesis that polyploidy enables large phenotypic leaps, providing access to many different therapy-resistant phenotypes. We develop a flow-cytometry based method for quantifying the prevalence of polyploid tumor cells, and show the frequency of these cells in patient tumors may be higher than is generally appreciated. We then present recent studies identifying promising new therapeutic strategies that could be used to specifically target polyploid tumor cells in cancer patients. We argue that these therapeutic approaches should be incorporated into new treatment strategies aimed at blocking tumor evolution by killing the highly evolvable, therapy-resistant polyploid cell subpopulations, thus helping to maintain patient tumors in a drug sensitive state.

## Coming to Terms with Cancer as a Rapidly Evolving System

It has long been appreciated that cancer is an evolutionary system (1). In this paradigm, individual cancer cells are the reproductive units within a tumor, with those cells that acquire a survival advantage through random genetic change being selected through multiple rounds of clonal expansion, during which they acquire further alterations that eventually combine to produce malignant phenotypes (1). The ability of a tumor to evolve solutions to selection pressures is a function of the *selectable heritable variation* that is present within the tumor, be it internal stressors such as low oxygen tumor micro-environments, or external stressors such as anti-cancer therapies (2–8). The paradigm of selectable heritable variation at the cellular level being a critical driver of cancer biology has been captured by the term *tumor heterogeneity*, and the emerging consensus is that tumor heterogeneity remains a fundamental obstacle preventing the development of truly curative anti-cancer therapies (2–8).

The introduction of efficacious targeted therapies highlighted the central role of evolution in cancer therapy failure. Patients with leukemia and lung cancer treated with specific inhibitors targeting oncogenic receptor tyrosine kinase (RTK) activity, eventually exhibit disease progression driven by point mutations within the oncogenic RTK that renders the tumor resistant to further therapy (9–11). Retrospective analysis revealed rare therapy-resistant mutants present in tumors prior to treatment initiation (12), confirming that in some cases targeted therapy selected for resistant clones that were already present within the tumor system. Melanoma offers a compelling case study of tumor evolution during targeted therapy. The identification of oncogenic mutations within the B-Raf kinase led to the development of specific inhibitors that initially display phenomenal clinical efficacy (13–16), which is swiftly followed by disease recurrence driven by rapidly evolving therapy resistance [reviewed in Ref. (17)].

Immunological based therapies are also vulnerable to therapy-resistance driven by tumor evolution, as revealed during a vaccine strategy trialed in adult patients with Glioblastoma. The vaccine therapy invokes a patient immune response that specifically targets the truncated, oncogenic EGFRvIII variant of the EGF receptor (18). The EGFRvIII variant is present in approximately one-third of Glioblastoma patients (19) and is an ideal target for anti-tumor immunotherapy because the constitutive activity of the EGFRvIII contributes to tumorigenicity, invasion and therapy resistance [reviewed in Ref. (18)]. Although the vaccine significantly increased overall survival time in treated patients whose tumors expressed the EGFRvIII receptor, disease recurrence occurred in all patients with most recurrent tumors losing EGFRvIII expression (18). EGFRvIII expression is typically heterogeneous in Glioblastoma tumors, and is only observed in a sub-population of tumor cells and rarely in the entire tumor (20, 21). The most plausible hypothesis is that the vaccine led to the immune-clearance of EGFRvIII expressing cells from patient tumors, but in the majority of cases it was the presence of viable EGFRvIII negative cells within the tumor that allowed immunological escape and rapid disease recurrence.

These and many other studies all converge on the hypothesis that long-term cancer patient survival requires the development of therapeutic strategies that actively suppress tumor evolution (2–8, 22). In this perspective, we propose that tumor cells containing an elevated genomic content (as described in Box 1) are key players in tumor evolution, and are therefore important therapeutic targets in preventing the acquisition of therapy resistance during treatment. We begin by summarizing seminal work conducted in yeast that characterizes how chromosomal gains facilitate rapid evolution under a wide variety of selection pressures. Next, we review recent work conducted in cancer, which show that chromosomal gains also underpin tumor initiation and the acquisition of therapy resistance in cancer patients. We then present an updated model of tumor evolution that highlights the central role of increasing ploidy in cancer initiation and disease progression.

Box 1 **Definitions.**

**Polyploidy:** An alteration of chromosomal number that is a multiple of the normal diploid (2n) complement.

**Tetraploidy:** A specific form of polyploidy that is a doubling of the normal diploid complement (i.e., 4n).

**Aneuploidy:** An alteration of chromosome number that is not a multiple of the diploid (2n) complement.

**Hyperdiploidy:** Having a chromosome number that is more than the diploid (2n) complement.

Around 90% of all solid tumors are aneuploid, and most aneuploidy tumors exhibit chromosomal gains and are therefore hyperdiploid (85). Many cancers have complex karyotypes [see for example Ref. (62)]. In this perspective, we have focused on subpopulations of cancer cells that have elevated genomic content relative to the tumor bulk, as a source of cells capable of rapid evolution. In the strict sense, these cells are grossly hyperdiploid relative to healthy, untransformed cells. However, we refer to them here simply as polyploid tumor cells (or pseudo-polyploid tumor cells) for the following reasons. (1) We are comparing these cells to the tumor bulk, and grossly hyperdiploid tumor sub-population of cells are typically polyploid (or close to polyploid) relative to the dominant aneuploid tumor karyotype, (2) many of the cited cell biology studies refer to this tumor cell sub-population as polyploid. We ask the reader to keep in mind that the “tumor polyploid cells” are in reality a genetically heterogeneous sub-population, which is composed of a variety of complex cancer karyotypes that are approximately polyploid relative to the dominant, aneuploid tumor cell population.

We finish the perspective showcasing recent studies that identify anti-polyploid compounds that we hope will provide a foundation for the development of efficacious chemopreventative and evolutionary suppressing cancer therapies of the future. Our goal is to focus research efforts on the development and translation of such novel anti-polyploid therapies to prevent and treat incurable cancers.

## Hyperdiploidy and Polyploidy Facilitates Rapid Evolution: Lessons from Yeast that are Relevant to Cancer

### How increasing genome size facilitates rapid evolution in yeast

Serious systemic fungal infections continue to endanger patients with immunocompromised immune systems (23, 24). Anti-fungal azole drugs are the most commonly used therapy against superficial and systemic fungal infections due to their efficacy and safety (23). Fluconazole is a widely used azole that is orally and intravenously available and effective against *Candida* infections, and is used clinically to treat oropharyngeal and esophageal *Candidas* in HIV patients, invasive candidiasis, as well as fungal infections in the urinary tract and central nervous system (23). Prior to the HIV pandemic, fluconazole resistance was rare. However, the widespread use of fluconazole to treat HIV/AIDS patients has increased the incidence of fluconazole-resistant *Candida* isolates (25). Generally, resistance develops after administration of sub-optimal doses of fluconazole over long periods of time, but in 1992, Bossche et al. isolated a resistant *Candida* strain in a patient after only 9 days of fluconazole treatment (26), revealing circumstances under which the evolution of fluconazole therapy-resistance occurs astonishingly quickly. In a follow-up study examining the mechanisms underlying the rapidly acquired fluconazole resistance, it was found that the resistant strain expressed more cytochrome P-450 14α-lanosterol demethylase (the target for azole antifungals) due to duplication of the entire chromosome containing the CYPO51 gene (27). Subsequent studies have confirmed that chromosome duplication is an effective and widely utilized mechanism to evolve drug-resistance in fungal infections (28–31).

Increasing chromosome numbers also provides fitness advantages in other contexts. A powerful example of rapid adaptation through increasing genomic content was provided by Rancati et al. (32) when they experimentally perturbed cytokinesis by deleting the MYO1 gene in the yeast *Saccharomyces cerevisiae*, and then selected for mutant strains that had evolved a solution to MYO1 deletion to restore functional cytokinesis. Strikingly, they found that most of the evolved strains, including the 10 fittest isolates, displayed an increase in DNA content. Further, diploid strains evolved much faster than haploid strains. Together, these data suggest that polyploidization facilitated the rapid evolution of cytokinesis rescue, a finding reminiscent of the rapid evolution of therapy-resistance driven by polyploidy described in immunocompromised patients treated with anti-fungal therapies described above.

Hyperosmotic stress occurs when an organism is exposed to higher solute concentration outside the cell, leading to water loss and subsequent increases in intracellular ion and metabolite concentrations [reviewed in Ref. (33)]. Hyperosmotic stress is a common environmental stressor, and yeast have evolved a hyperosmotic stress response that is mediated by the high osmolarity glycerol (HOG) pathway, which activates genes involved in salt tolerance and adaptation (34). Wagner and colleagues investigated how yeast could evolve adaptations to hyperosmolarity stress in long-term evolution experiments, where three replicate *Saccharomyces cerevisiae* yeast populations were exposed to high-salt conditions for 300 generations (33). All three populations evolved a faster growth rate under high-salt conditions after selection compared to their ancestral cultures (33). DNA content analysis revealed that all three evolved lines had an increase in ploidy, suggesting that evolutionary adaptation to hyperosmotic stress is also facilitated through increasing genome size (33).

The Evolution Canyon originated in Israel 3–5 million years ago, and contains diverse micro-environments and has experienced minimal human disturbance, providing an excellent natural site to study evolutionary adaptations of many organisms (35). Chang et al. isolated and phenotypically characterized 14 diploid yeast strains collected from different micro-environments present within the Evolutionary Canyon (35). One of these strains was highly resistant to the metal copper. Strikingly, Chang et al. found that the copper-tolerant phenotype was the product of large-scale chromosomal rearrangements that increased the copy number of the CUP1 and CUP2, major genes involved in copper regulation (35). Additional copper-tolerance gene expression was up regulated by increased CUP2 copy number, showing that the increase in gene dosage both directly and indirectly contributes to the evolution of copper-tolerance. Surprisingly, when the tolerant strains were cultured in the absence of copper, a wild-type chromosome reappeared and was fixed within 300 generations. These findings reveal that “large-scale chromosomal rearrangements provide not only fast arising but also readily reversible sources of variation during early stages of adaptive evolution” (35).

Collectively, these studies reveal increasing chromosome content as a mechanism that facilitates the rapid evolution of yeast across many different selection pressures and environments. These include the rapid acquisition of therapy resistance in patients, rapid adaptation during experimental evolution, and the successful adaptation to selection pressures present in nature.

### How increasing genome size changes yeast phenotypes

One important mechanism for rapid adaptation provided by chromosomal gain is increased gene expression due to elevated gene dosage. Multiple studies have confirmed that messenger RNA levels scale with chromosome copy number in aneuploid systems. Hughes et al. conducted expression profiling of yeast strains with characterized aneuploidy and showed that increased genomic content data “precisely mirrored the expression data in this region” (36), revealing that gene duplication leads to a commensurate increase in messenger expression (36). The second important finding from this study was that under experimental selection, large-scale gene duplications were shown to be the dominant adaptive response to loss-of-function deletions (36), providing early support for the hypothesis that increasing genomic content facilitates rapid adaptation.

In a later study, examining the effects of extra chromosomes on cell physiology and cell division in yeast, Torres et al. observed an approximate doubling of gene expression in duplicated chromosomes, with greater than 90% the amplified genes being expressed at a higher level (37). These data indicate that most genes are expressed in proportion to their gene copy number, and gene amplification results in a roughly proportional increase in gene expression (37).

The classic evolution study by Rancati et al. confirmed that on average there is a stoichiometric relationship between gene copy number and gene expression level, with gene expression levels from chromosomes roughly scaling with chromosome copy number (32). However, they noted that some gene expression levels deviated significantly from this trend, identifying outlier genes whose expression changed more than three standard deviations away from the stoichiometric trend (32). Further evidence suggests that the majority of outlier expression is caused by the increased expression of transcription factors (or their upstream regulators) caused by chromosomal copy number increase (32). Similarly, expression of the copper resistance gene CUP2 due to increase in gene dosage causes the expression of downstream genes, several of which also enhance resistance to copper (35). This reveals how a simple linear change in gene expression can generate a non-linear adaptive response through pathway amplification (35).

Changes in yeast chromosome numbers also increase protein expression levels in yeast cells. Pavelka et al. generated a panel of stable aneuploidy yeast strains to directly address this question (38). They found that chromosomal copy number changes in general caused proportional changes in gene expression and protein expression levels (38). Further, they found that yeast strains with similar karyotypes tend to display similar changes in global protein expression patterns (38). Interestingly, the Authors also identified outliers in gene and protein expression, however only a small fraction of the gene expression outliers overlapped with the protein expression outliers (38), revealing that gene dosage changes are likely to have complex effects on cellular phenotypes. The Authors applied a variety of selection pressures on euploid parent controls and aneuploid strains, revealing that aneuploid strains grew better under selection by generating rapid phenotypic variation, showing that aneuploidy that can provide fitness gains under diverse selection pressures (38).

Together, the data sets summarized above show that increasing DNA content modifies both gene and protein expression, in linear and non-linear ways, allowing cell populations to rapidly explore a wide range of heritable phenotypes. Thus, increasing ploidy enables yeast cells to experience large phenotypic leaps, which in turn facilitates rapid evolution to novel selection pressures (39).

### How increased genomic content buffers cells against deleterious mutations

The mutator hypothesis proposes that mutations that increase genomic instability (the mutator phenotype) drives tumorigenesis by allowing cells to rapidly acquire the necessary number of mutations required for cellular transformation (40). The mutator phenotype was first proposed by Loeb to explain how tumors can accumulate the number of mutations necessary for tumorigenesis despite the extremely high accuracy with which mammalian cells replicate the genome (41, 42). One primary criticism of the mutator hypothesis is that most mutations are deleterious and therefore the mutator phenotype will accelerate the accumulation of mutations that reduce fitness, leading to negative clonal selection [(3) and references therein]. Although experimental and theoretical counters to this criticism have been provided [recently reviewed in Ref. (43)], one potentially important phenomenon that has been overlooked in this debate is the role of genome amplification in buffering eukaryotic cells against the effects of deleterious mutations.

Using adaptation to different laboratory environments as their selection pressure, Thompson et al. (44) compared the relative fitness of mismatch repair defective (mutator) strains of yeast within haploid and diploid yeast genetic backgrounds, with striking results. In the diploid genetic background, mutators displayed an advantage over non-mutators, and mutators that “win” adaptation experiments were on average fitter than non-mutator winners (44). In contrast mutators in the haploid background displayed no advantage when competed against haploid non-mutators and the haploid mutator winners were less fit than the haploid non-mutator controls (44). The most parsimonious explanation for this result is that most deleterious mutations are recessive, and are therefore buffered in the diploid yeast strain. Haploid yeast must bear the cost of deleterious mutations in full, which gives haploid yeast less time to accumulate beneficial mutations before the cumulative effects of deleterious mutations eliminates them from the population. An additional important observation from this study was the type of mutations that occurred with haploid versus diploid populations. The diploid mutators displayed a generalist class of beneficial mutation that provided a large selective advantage across a range of selection pressures (44). In contrast, haploid mutators displayed beneficial mutations whose advantage was limited to the specific stress they were selected under (44).

These results suggest two intriguing hypotheses. First, increases in ploidy may act co-operatively with a mutator phenotype by reducing the effect of deleterious mutations. Second, increased ploidy enables a mutator phenotype to generate “generalist” beneficial mutations that confer selective advantage across a wide range of stressors.

## How Elevated Ploidy Drives the Evolution of Cancer

### Aneuploidy and tumorigenesis

The vast majority of cancers are aneuploidy, with around 90% of solid tumors and 75% of hematopoietic cancers having abnormal chromosome numbers (45). The high incidence of aneuploidy in cancer cells inspired Boveri over 100 years ago to propose the hypothesis that aneuploidy causes cancer (46). Consistent with this hypothesis, aneuploidy has been shown to precede transformation in a variety of cancers (47–54), and several studies provide both experimental and theoretical support for a fundamental role of aneuploidy during tumor initiation (55–59). Duesberg and colleagues have proposed that aneuploidy generates cancer causing karyotypes that are selected during the evolutionary process of tumor initiation and transformation (60–62). However, these ideas have been contested, in part because the aneuploidy model of tumor initiation is thought to downplay the established role of oncogenes in the process of transformation (63–65). Recent studies examining the role of polyploidy in tumor initiation may help incorporate the oncogenes and aneuploidy tumor initiation models into a single paradigm.

### Evidence supporting tetraploid cells being the cell of origin in tumor initiation

Experimental evidence directly linking tetraploidy with tumor initiation was provided when Fujiwara et al. created a tetraploid cell population in p53-null mouse mammary epithelial cells (66). Tetraploid cells displayed a high level of tumorigenesis when injected into nude mice, in contrast to the diploid p53-null controls, which did not form tumors (66). Subsequent studies perturbing the mitotic spindle led to accumulation of tetraploid cells and a higher incidence of tumor formation, further supporting a central role of tetraploidy in tumor initiation (67, 68).

Tetraploidy potentially provides multiple beneficial functions during tumor initiation. First, a large body of evidence supports the hypothesis that tetraploidy acts as a gateway karyotype by inducing chromosomal instability (CIN), which leads to aneuploidy and the evolution of a transformed phenotype [reviewed in Ref. (69)]. Using several experimental models of telomere crisis, Davoli and de Lange recently demonstrated that endoreplication and mitotic failure created tetraploid cells during telomere crisis (70). Importantly, the resulting tetraploid cells displayed enhanced tumorigenic capacity relative to diploid controls in soft agar and mouse implantation assays (70). Finally, Davoli and de Lange then showed tumors that are initiated by tetraploid cells evolve more complex aneuploidy karyotypes *in vivo*, showing tetraploidy functions as a gateway mutation to aneuploidy (70).

Lv and colleagues used the spontaneous transformation of primary ovarian epithelial cells to provide compelling evidence for the role of tetraploidy as a gateway karyotype during tumorigenesis (71). Lv et al. generated primary cultures of mouse ovarian surface epithelial cells (MOSECs), which they continually subcultured for over 30 passages (71). Following ploidy status during culture revealed that the diploid cells underwent an intermediate tetraploid phase, and then evolved into aneuploid (near-tetraploid) cells (71). Tetraploidy was caused by cytokinesis failure in diploid cells, with the tetraploid cells subsequently experiencing chromosome mis-segregation during bipolar and multipolar mitosis to generate aneuploid progeny (71). When the lines were re-injected into mice, only late passage aneuploid cells formed tumors (71), showing that spontaneous transformation during long-term passaging likely involves a diploid–tetraploid–aneuploid transition caused by defects in mitosis.

Two recent studies have provided compelling support for the hypothesis that genome doubling facilitates the acquisition of a transformed phenotype in tumor initiation in human cancers. Examining neuroblastomas, Lundberg et al. combined karyotypic analyses of tumors with mathematical modeling and concluded that the loss of chromosomes from a tetraploid precursor cell was the most parsimonious hypothesis explaining the chromosomal numerical alterations present in neuroblastoma tumors (72). This conclusion was supported experimentally when it was shown that neuroblastoma lines displayed a high frequency of polyploidization events, and that clonal cultures with elevated genomic content generated aneuploid progeny with high frequency (72). Altogether these data suggest that polyploidy is a gateway cell state that facilitates the generation of aneuploidy and increases karyotypic complexity in neuroblastoma tumors (72).

More recently, Swanton and colleagues (73) systematically addressed the role of tetraploidy in colorectal cancer evolution (73). Colorectal cancers that had undergone genome doubling (i.e., tetraploid) displayed a significantly higher incidence of genomic instability than those cancers that began as diploids, with tetraploidization appearing to be an early event in the majority of colorectal cancers (73). Tetraploid clones were isolated from colorectal cancer lines, and these displayed a higher incidence of segregation errors during anaphase and increased chromosomal structural abnormalities relative to their cognate, diploid controls (73). Strikingly, daughter cells derived from diploid clones that had undergone a segregation error during mitosis frequently died or underwent cell-cycle arrest, whereas daughter generated from tetraploid clones after segregation error died much less frequently and continued to proliferate (73). These data provide direct experimental support for the hypothesis that tetraploidy endows tumor precursor cells with an elevated tolerance to CIN, facilitating the generation of aneuploidy and the evolution of a complex karyotype (74). Consistent with this model, genome doubling is associated with poor prognosis, being significantly associated with disease relapse (73).

In addition to increasing tolerance to aneuploidy and facilitating the evolution of a transformed karyotype, tetraploidy also helps overcome oncogene induced senescence. Aberrant activation of oncogenes such as Ras, Raf, or PI3-kinase triggers cellular senescence, which functions as a tumor suppressor by permanently restricting the proliferative capacity of cells (75–77). Activation of DNA-damage response pathways plays an important role during oncogene induced senescence (78–80), as does activation of p53 pathways (76, 81–83). Exploring how malignant cells overcome the senescent barrier, Zheng et al. used a mouse model of tumorigenesis discovered that cells that overcame tumorigenesis barriers to drive long-term proliferation in culture all displayed near-polyploid levels of aneuploidy (84). These near-polyploid cells overexpressed DNA repair genes to reduce the DNA-damage response, as well as methylating p53 promoter regions to silence p53 expression (84). These results indicate that polyploid cells may be able to overcome the oncogene induced senescence by increasing DNA repair activity and epigenetic reprograming of p53 expression (84).

Altogether, these studies show that tetraploidy functions as a gateway phenotype that cooperates with oncogenes to induce cellular transformation in three ways. First, tetraploidy helps overcome oncogene induced senescence. Second, tetraploidy facilitates the acquisition of oncogenic karyotypes and phenotypes by inducing CIN leading to aneuploidy. Third tetraploidy buffers pre-malignant cells against the deleterious effects of chromosomal loss. Collectively, these findings go some way to explaining why the majority of human tumors contain a hyperdiploid karyotype (85).

## Polyploidy Tumor Cells and the Evolution of Cancer Therapy Resistance

### How polyploidy overcomes therapy-induced senescence

Cancer cells can survive chemotherapy and radiotherapy by entering a reversible senescent state, called therapy-induced senescence (TIS), which is a senescent-like phenotype that displays many of the features of the normal physiological senescence phenotype (86). Even transformed cells lacking functional p53 and retinoblastoma protein (Rb) pathways retain the capacity to undergo TIS (87). TIS has been observed *in vivo* using both xenograft and transgenic cancer models (88, 89). Senescence markers have been observed from breast and lung cancer patient tumor specimens treated with chemotherapy, supporting the hypothesis that TIS is a clinically relevant cell fate in human cancer patients treated with cytotoxic therapies (90, 91).

Unfortunately TIS is not permanent, with rare cells being able to bypass TIS to re-enter the cell cycle and re-initiate tumor growth (90). One way cells overcome TIS is through the over-expression of the mitotic kinase CDK1, which phosphorylates the protein survivin to promote TIS escape and subsequent survival of cancer cells (92). In a follow-up study, Wang and colleagues went on to show that over-expression of CDK1 induced the formation of polyploid cells during TIS, and that these CDK expressing polyploid cells represent an important transition state through which escape from TIS preferentially occurs (92). Intriguingly, Wang et al. also reported that non-small cell lung cancer patients expressing markers of TIS following neo-adjuvant therapy had a significantly worse prognosis than patients who did not express TIS markers (92). Altogether, these data support a model whereby TIS provides an escape mechanism for tumor cells to avoid the toxic effects of chemotherapy to drive disease recurrence (92). Moreover, polyploid tumor cells are far more likely to overcome the TIS barrier, and polyploidy-mediated TIS escape represents an important new therapy-resistance mechanism in cancer patients undergoing a variety of chemotherapy regimes (92).

### How polyploidy induces infrequent cell cycle

Infrequent cell cycle is a well-established resistance mechanism against cytotoxic insult. Normal quiescent (G_0_) hematopoietic stem cells (HSCs) are resistant to the anti-proliferative chemotherapeutic agent 5-fluoro-uracil (5-FU) (93, 94), and become sensitive to 5-FU treatment when they are forced into a proliferative state by treatment with IFNα (95). Healthy HSCs can be protected from the effects of irradiation by increasing the proportion of HSCs in G_0_ through a variety of treatments *in vivo* (96–98). In cancer, the chemoprotective effect of cell-cycle-mediated drug-resistance is well-established (99). For example, Schmidt and colleagues demonstrated that colon adenocarcinoma cells arrested in G_1_ by over-expression of p27^Kip1^ are significantly more resistant to a variety of chemotherapeutic agents, including temozolomide (100). Using a mouse xenograft model, Naumov et al. showed that the DNA intercalating compound doxorubicin (DXR) effectively reduced the metastatic tumor burden but spared non-cycling tumor cells, which persisted during therapy and subsequently developed into metastases after DXR therapy was discontinued (101). More recently, label-retention has been used to phenotypically identify infrequently dividing cells that are resistant to chemotherapy from a variety of tumor types (102–105). Studies examining the cancer stem-cell phenotype have also shown that quiescence provides protection against cell death induced by DNA-damage agents (106, 107) and chemotherapy (108). Recently, a landmark study by Kreso et al. revealed how chemotherapy selects for minor, infrequently cycling subpopulations using lineage tracking in mouse models of cancer evolution (109). Collectively these studies provide strong support the hypothesis that infrequent cell cycle as a fundamental mechanism that contributes to the evolution of therapy resistance in cancer patients.

Recently, we identified a genetically diverse, polyploid tumor cell sub-population in Glioblastoma patients that is able to initiate and maintain tumor growth *in vivo*, and is resistant to cytotoxic therapy (110). Proliferation markers revealed that the polyploid tumor cell sub-population contain approximately three times more quiescent cells than the bulk near-diploid tumor population (110). Infrequently cycling cells retain the dye CFSE, and CFSE label-retention has been used to enrich for therapy-resistant, tumor-initiating cells in several tumor types [reviewed in Ref. (111)]. Polyploid tumor cells accumulate within the label-retaining sub-population of cells, providing a functional confirmation of their infrequent cell cycle (110). Altogether, these data show that increasing chromosome numbers provides a mechanism to generate infrequently cycling tumor cells, providing a general resistance mechanism against cytotoxic chemotherapy treatments designed to target actively cycling cells (110).

Why do polyploid tumor cells cycle less frequently? Seminal studies conducted in yeast show that increased transcription and translation caused by elevated genomic content causes cell-cycle delays during G_1_ (37). Murine embryonic fibroblasts (MEFs) containing extra chromosome copies also cycle less frequently (112, 113), likely due to changes in transcription and translation (85). In addition, polyploid tumor cells have a twofold larger cell volume compared to their diploid counterparts (110). Cell growth, cell size, and cell division are co-regulated to ensure cells are large enough to divide at mitosis (114). Studies in yeast reveal a size requirement for G_1_-S transition, with smaller cells delaying in G_1_ until a sufficient size was reached to maintain viable progeny after cell division (115, 116). Complementary studies in animal cells show that mammalian cells also delay in G_1_ to allow an appropriate cell size to be achieved (117, 118). A plausible hypothesis that combines both these observations is that the larger polyploid tumor cells arrest during G_0_/G_1_ to allow for a sufficient growth to occur before committing to division, which is hampered due to the increased transcription and translational demands placed on polyploid tumor cells by their elevated and unbalanced chromosomal copy number.

Thus increased ploidy provides a general resistance mechanism (that of infrequent cell cycle) to tumor cells, which are well-positioned to contribute to the rapid evolution of patient tumors during conventional chemotherapy and radiotherapy regimes.

### The role of giant polyploid cells in therapy resistance and tumor repopulation after therapy

Giant polyploid cells are formed if DNA replication is uncoupled from mitosis (119). This process has been termed the endocycle and is a characteristic of p53-null cells (120), which is further increased by exposure to radiation (121). It was thought that the process of endocycles was irreversible and the resulting giant polyploid cells represent a reproductive dead end (122). However two back-to-back manuscripts suggested that giant polyploid cells may provide an escape mechanism from severe genotoxic damage. The first study followed p53-null cells after genotoxic insult, noting that after delaying at G2/M for several days the cells enter endoreplication cycles that generate giant polyploid cells (123). Although the majority of giant polyploid cells die, a small subset survive that are able to produce viable progeny cells as determined using sensitive clonogenic assays (123). Viable giant polyploid cells appear to follow a defined path of chromosome re-organization that involves reconstructing nuclei into polyploidy “bouquets,” which subsequently return to an interphase state and separate into secondary nuclei (124). These secondary nuclei give rise to secondary cells in a manner reminiscent of the life-cycles of protozoa (124).

Looking at two forms of transformation, carcinogen-induced transformation of p53^+/+^ cell lines and spontaneous transformation of p53^−/−^ cell lines Sundaram et al. reported a transformation process that involved giant polyploid cell intermediates (125). Here, the giant polyploid cells undergo a novel type of cell division that involves nuclear budding within the giant polyploid cells followed by intracellular cytokinesis to produce mononuclear daughter cells that bud off the parental giant polyploid mother cells (125). These mononuclear daughter cells are transformed, displaying anchorage-independent growth (a classical hallmark of cellular transformation) (125). A series of follow-up studies provided strong support for the hypothesis that a subset giant polyploid cells undergo some form of reductive division to produce small cells with near-diploid chromosomes that are proliferative and competent to re-initiate tumor growth (reviewed in Ref. (126)]. Interestingly, irradiated giant polyploid cells activate key meiotic genes that are involved in metaphase arrest, genetic recombination, and reductive divisions that occur during meiosis, indicating that giant polyploidy reductive divisions are likely “meiosis-like” in nature (127–130).

Puig et al. undertook a systematic study using xenograft *in vivo* models and *in vitro* approaches to characterize the role of giant polyploid cells in therapy response to cisplatin (131). Cisplatin treated tumors initially undergo shrinkage, and are increasingly populated with giant non-proliferating tumor cells that maintain DNA synthesis (131). After several weeks of latency tumor growth recurs, driven by a small fraction of proliferating cells (131). Cells treated *in vitro* using clinically relevant cisplatin doses also generate giant polyploid cells, a subset of which are able to generate colonies of rapidly cycling small diploid cells. This recapitulated the *in vivo* disease recurrence and suggested that giant polyploid cells are active contributors to disease progression after therapy (131). Intriguingly, the proliferative diploid cells generated from giant polyploidy cells have altered karyotypes and display increased resistance to cytotoxic drugs (131), suggesting for the first time that giant polyploid cells actively contribute to the evolution of therapy resistance.

Very recent work studying ovarian cancer has underscored the importance of giant polyploid cells in cancer disease progression and therapy resistance (132). Zhang et al. purified giant polyploid cells from established ovarian cancer lines and patient tumors, and confirmed that these cells can initiate tumors *in vivo* and are resistant to cisplatin cytotoxic therapy (132). Like previous studies, Zhang et al. confirmed that giant polyploidy cells cycle infrequently and generate smaller near-diploid progeny through budding and bursting mechanisms (132). In this way, giant polyploid cells are posited to function in a manner analogous to spores in lower organisms, surviving harsh conditions to facilitate rapid repopulation after stressful conditions have subsided (132, 133).

### Polyploidy, EMT, and the cancer stem-cell phenotype

Cells with a primitive, undifferentiated phenotype tend to cycle infrequently and display enhanced DNA repair, making them difficult to kill using cytotoxic and genotoxic therapies that preferentially target actively cycling cells (134–136). The underlying drivers leading to the generation of a primitive phenotype in patient tumors remain incompletely understood. It has been reported that the frequency of CSC’s increases after treatment with genotoxic therapies (137–139). Salmina et al. tested the hypothesis whether polyploidy, which allows cells to survive cytotoxic therapy to continue proliferation, is also capable of endowing cells with a primitive cell phenotype (140). They found that irradiated giant polyploidy cells caused up regulation of the self-renewal stem-cell genes OCT4 and NANOG, and that the NANOG, OCT4, and SOX2 proteins were concentrated onto nuclear foci in giant polyploidy cells (140). The giant polyploid cells resisted apoptosis, overcame TIS, and transmitted the NANOG-OCT4-SOX2 self-renewal program to their progeny (140). Subsequently Lagadec et al. reported that ionizing radiation reprogramed differentiated breast cancer cells toward an undifferentiated CSC state (141). Strikingly, CSC reprograming only occurred within polyploidy subpopulations, and involved re-expression of the transcription factors OCT4, NANOG, sex determining region Y-box 2 and Klf4 (141). More recently, Zhang et al. demonstrated that ovarian cancer giant polyploid cells displayed the CSC properties of CD44^+^/CD133^+^ expression, generation of spheroids under serum-free culture conditions, increased tumorigenicity, and elevated therapy resistance (132).

Cancer cells can also undergo epithelial to mesenchymal transition (EMT), where the cancer cells activate an evolutionarily conserved trans-differentiation program that is used during morphogenesis to convert differentiated epithelial cells into migratory mesenchymal cells [reviewed in Ref. (142)]. Cancer cells undergoing EMT not only adopt an invasive cell phenotype that can drive metastasis, but may also enter a drug refractory state due to epigenetic reprograming (142). Recent work has revealed that polyploidy facilitates EMT, with Zhang et al. showing that giant polyploid tumor cells gain a mesenchymal phenotype (132) that correlates with increased expression levels of EMT transcriptional factors (143). These data suggest that polyploidy can facilitate EMT, providing access to cell phenotype that is both invasive and resistant to a variety of therapies.

Together, these studies provide compelling support for the hypothesis that polyploidy drives the acquisition of undifferentiated, primitive cellular phenotypes in human cancer. These cell phenotypes can potentially increase therapy resistance, provide an elevated tumor initiation capacity, and increase both the invasive and metastatic potential of tumor cells.

### Ploidy-induced escape from targeted anti-cancer therapies

Strong evidence supporting the role of polyploidy in evolving solutions to targeted therapy has come from mouse models of cancer. A defective spindle assembly checkpoint (SAC) results in “mitotic slippage,” where cells exit mitosis without undergoing anaphase or cytokinesis to produce a tetraploid cell [reviewed in Ref. (69)]. As essential component of the SAC is Mad2, and Mad2 over-expression commonly occurs in many human cancers and is associated with poor prognosis [reviewed in Ref. (68)]. Over-expression of Mad2 increases the frequency of mitotic slippage and tetraploidy (68, 69), and promotes tumorigenesis in mice (69). In a doxycycline-inducible K-Ras model of cancer, Sotillo and colleagues explored how Mad2 over-expression determined the tumors ability to escape inhibition of the primary oncogenic driver K-Ras (144). In these experiments, Sotillo et al. allowed K-Ras tumors to form in the presence or absence of Mad2, revealing that the presence of Mad2 expression increased the aggressiveness of the K-Ras tumor, as indicated by increased invasion, elevated proliferative index, and a significant decrease in overall survival (144). When doxycycline was removed, K-Ras and Mad2 expression was lost, leading to tumor regression in all animals. K-Ras only tumors recurred rarely, however the tumors expressing both K-Ras and Mad2 displayed a marked increase in recurrence rate, driven by activation of a variety of compensating transforming pathways (144). This finding supports the hypothesis that CIN increases the probability of disease relapse during targeted therapy by facilitating alternate pathway activation that allows tumor cells to avoid the effects of targeted therapy (144). Further, this study highlights how aneuploidy and oncogenes can act synergistically during tumor initiation and cancer evolution.

The proteasome inhibitor, bortezomib, has forged new horizons in the treatment of multiple myeloma (MM) (145). Although efficacious, bortezomib is non-curative for MM because patients eventually evolve therapy resistance. However, the underlying resistance mechanisms remain poorly understood (146). To begin to characterize resistance mechanisms, Balsas et al. generated bortezomib-resistant MM lines that displayed five to sixfold increased resistance to bortezomib (147). Unexpectedly, the target of bortezomib (PSMβ5, the β5 subunit of the proteasome) was not mutated, but was instead significantly overexpressed at both the mRNA and protein levels within resistant cells (147). In addition, the bortezomib-resistant cells had evolved a near-tetraploid genomic content, which also displayed cross-resistance to other chemically unrelated proteasome inhibitors (147). Together, these data provide direct support for the hypothesis that, as for yeast, increasing genomic content allows cancer cells to circumvent targeted therapy through over-expression of the therapy target.

An interesting and unwelcome twist to the use of targeted therapy came from study of Sharma et al. (148). When Sharma et al. treated several breast cancer lines with the tyrosine kinase inhibitor BMS-777607, they noted that the surviving cell population displayed elevated levels of polyploidy due to an increase in the incidence of failed cytokinesis caused by off-target inhibition of Aurora kinase B (148). They tested the surviving polyploidy cells for sensitivity toward a range of chemotherapeutics (doxorubicin, bleomycin, cisplatin, methotrexate, and paclitaxel), and found that the therapy-induced polyploidy cells were resistant to all classes of chemotherapies tested (148). This finding is reminiscent of an evolutionary study undertaken in yeast, where transiently targeting the function of Hsp90 protein led the chromosomal gains and the rapid evolution of therapy-resistance toward unrelated cytotoxic compounds (149).

Together these studies reveal that tumor cell polyploidy generates resistance toward targeted therapy. Of concern is the finding that treating tumor cells with targeted therapies can elevate levels of polyploidy in tumor cell populations, which then increases the risk of developing multi-drug-resistance within clinical settings.

### Measuring the prevalence of polyploid tumor cells in cancer

How many polyploid cells are there in patient tumors? Quantitation of polyploidy in patient cell lines and primary tumors is challenging due to the infrequent cell cycle of polyploid tumor cells. Cancer cell biologists have traditionally used a flow-cytometry approach, where they estimate the frequency of polyploidy by measuring the number of cells with greater than 4n DNA content. However, this approach can only detect polyploid cells that are actively cycling, because most of the polyploid tumor cells are tetraploid or near-tetraploid, and therefore remain indistinguishable from the G_2_/M cells of the “diploid” tumor population. More sophisticated metaphase analyses [for example those in Ref. (62)] are also likely to underestimate the frequency of polyploid tumor cells, because their infrequent cell cycle means they will be under-represented using a metaphase-dependent karyotypic analysis.

**Figure 1:**
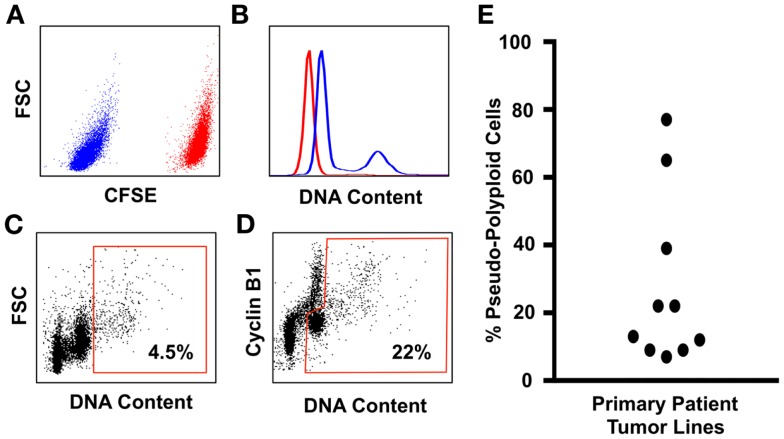
**An improved flow-cytometry assay for measuring the prevalence of polyploidy in tumors cell populations**. **(A)** Tumor cell samples are spiked with carboxyfluorescein diacetate succinimidylester (CFSE) stained primary neonatal foreskin fibroblasts (NFF) diploid control. The CFSE-negative tumor cells shown in blue are readily gated from the CFSE-high NFF diploid controls, shown in red. **(B)** DNA content of the Glioblastoma tumor cells (blue histogram) overlayed onto the NFF diploid control histogram, shown in red. Most Glioblastoma cell lines that we have studied are aneuploid with a slightly hyperdiploid DNA content, and contain a small sub-population of cells that are near-tetraploid with respect to the tumor bulk population (i.e., pseudo-polyploid). **(C)** A typical polyploidy flow-cytometry assessment utilized by many cancer cell biologists, who use the proportion of live single cells with greater than 4n DNA (shown within the red gate) as being representative of the total pseudo-polyploid population. In this example, 4.5% of the total cells are classed as pseudo-polyploid. **(D)** The same tumor sample assessed for pseudo-polyploidy using Cyclin-B1 staining to discriminate between the G_2_/M (the Cyclin-B1 *high* cells with a 4n DNA content) population of the pseudo-diploid bulk, from the pseudo-diploid G_0_/G_1_ population (the Cyclin-B1 *low* cells with a 4n DNA content). The pseudo-polyploid gate (shown in red) identifies both the cycling *and* the non-cycling pseudo-polyploid tumor cells, which make up approximately 22% of the total tumor cell population. **(E)** Ten low-passage primary patient glioblastoma cell lines, grown under serum-free tumorsphere conditions, assessed for pseudo-polyploidy using the Cyclin-B1 gating strategy from **(D)**. In eight lines, the dominant cell population was aneuploidy with a near-diploid DNA content, with a sub-population of pseudo-polyploid cells that made up 5–38% of the total cell population. In contrast, two glioblastoma lines were pre-dominantly pseudo-polyploid (65 and 78%), with a small of near-diploid sub-population. Detailed staining protocols are provided in Ref. (110).

Flow-cytometry screens can utilize the expression of Cyclin-B1 to discriminate between the cycling tumor “diploid” cells that are transitioning through the G_2_/M phase of the cell cycle, from the polyploid tumor cells in that are in the G_0/_G_1_ phase of the cell cycle (Figure 1). The advantage of this assay is that it allows the assessment of large populations of cells (in this example, 100,000 single cells for each patient line were analyzed), and the inclusion of a control line in the same tube means that the ploidy levels of tumor cells relative to control cells can be estimated within the same assay under identical staining conditions, eliminating the confounding effects of cell number variation between tubes [Figure 1; Ref. (110)]. Here, we use the prodrug carboxyfluorescein diacetate succinimidyl ester (CFSE), which is converted by cellular esterase activity into a fluorescent compound covalently bound to proteins and retained within the cells (150). CFSE staining clearly delineates the CFSE-stained control from the unstained test cell populations, and the control and test cell populations are readily identified using standard flow-cytometry gating strategies (110). CFSE-stained control lines can be either healthy diploid cells to provide a more accurate estimate of DNA content [(110) and shown in Figure 1], or alternatively untreated tumor cells can be used to directly compare the effect of drug treatments on the prevalence of polyploidy during compound screening or pre-clinical testing. Because control cells are stained with CFSE immediately before fixation (110), the staining process has no effect on cell ploidy or viability and is therefore unlikely to generate Type I or Type II errors during compound screening.

Using this method, we assessed the prevalence of polyploidy in 10 low-passage primary patient glioblastoma lines (Figure 1), cultured under tumorsphere conditions, a culture method that preserves the genotype, and phenotype of the original tumor (151). In 10 primary patient tumor lines, the lowest frequency of tumor cell polyploidy was 1 in 20 cells (i.e., 5% of the total tumor cell population were polyploid). To put this into context, it is estimated that a 1 g solid tumor contains 10^8^–10^9^ tumor cells (74, 152). If the lowest polyploid estimate of 5% is applied, then between 5 and 50 million rapidly evolving, therapy-resistant polyploid tumor cells will be present in brain cancer patients that have tumor volumes of 1 cm^3^.

## An Integrated Model Explaining How Increased Genomic Content Facilitates Cancer Evolution

**Figure 2:**
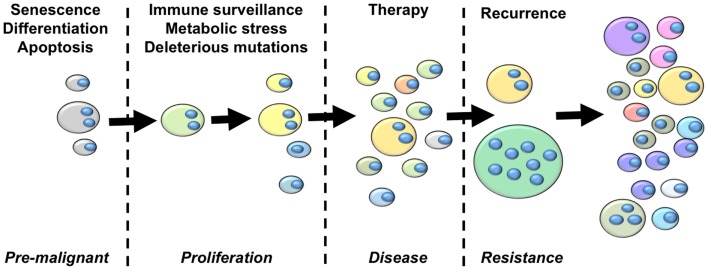
**An integrated model of tumor evolution highlighting potential roles of polyploidy during cellular transformation**. Here, we present a simplified view of disease progression, highlighting the role of polyploidy in overcoming selection pressures to drive the evolution of cellular transformation. The fist selections pressures pre-malignant lesions must overcome are those of apoptosis, senescence and terminal differentiation. Polyploidy enables adaptation to these barriers by silencing p53 remove p53-dependent pro-apoptotic and senescence signaling, rewiring the DNA-damage response to suppress p53-independent senescence programs, and enabling acquisition of primitive stem-cell phenotypes. Once a proliferative state is reached, polyploidy increases the acquisition of transforming mutations by increasing chromosomal instability and buffering the proliferative cells against the effects of deleterious mutations. Polyploidy also increases glycolysis, enabling survival in low oxygen environments, and enables EMT and the generation of invasive and metastatic phenotypes. Polyploid cells provide multiple mechanisms of therapy resistance, buffer the cancer genome against deleterious mutation resulting from genotoxic therapies, and generate primitive tumor-initiating phenotypes that are capable of driving disease recurrence. Throughout this process, tumor heterogeneity and karyotypic complexity increases, which in turn increase the heterogeneity and evolutionary capacity of the tumor.

From the perspective of cancer as an evolutionary disease, we argue that the studies summarized above provide sufficient grounds for the development of an updated model of cancer that highlights a central role of polyploidy during tumorigenesis and disease progression (Figure 2). The hallmarks of cancer outlined by Hanahan and Weinberg (153) clearly highlight the selection pressures that must be overcome on the journey from pre-malignant lesion to full-blown cancer. The early selection pressures include apoptosis, senescence and terminal differentiation. We argue that pre-malignant polyploid cells are more likely to overcome these barriers than diploid pre-malignant cells. Polyploidy enables epigenetic silencing of p53 (84), reducing the probability of apoptosis and weakening the senescence barrier. Polyploidy also rewires the DNA-damage response (84), further subverting the senescence barrier and increasing the probability of pre-malignant polyploid cells re-entering the cell cycle (84). The vast majority of cells are terminally differentiated. Differentiated pre-malignant cells must somehow overcome the terminal differentiation program, revert to an undifferentiated phenotype, and reclaim the unlimited proliferative capacity of multi-potent stem cells (154). We now know that polyploidy facilitates acquisition of a primitive, stem cell like phenotype (132, 140, 141), although the underlying mechanisms remain incompletely uncharacterized.

Once pre-malignant cells circumvent these initial selection pressures to generate a proliferative phenotype, they must then acquire further transforming mutations to overcome subsequent selection pressures such as immune-surveillance, metabolic stressors, and the effects of deleterious mutation (154). Polyploidy is likely to facilitate the rapid acquisition of new transforming mutations in two ways. First, elevated ploidy reduces the lethality of deleterious mutations and chromosome loss (44, 73, 74). Second, polyploidy increases CIN (70, 71, 73, 141, 155, 156), which elevates karyotypic variation within the tumor cell population through large-scale genetic change (74). Hence, polyploidy enables both CIN and mutator phenotypes, thereby greatly increasing the speed at which proliferating tumor precursor cells can acquire the portfolio of mutations and the oncogenic karyotypes necessary for full-blown transformation (3, 61). In addition, polyploidy can help overcome metabolic stress by contributing to metabolic reprograming, invasion, and metastasis. Polyploid tumor cells display elevated levels of anaerobic glycolysis (110, 157) and are highly resistant to oxygen deprivation (132). Polyploid tumor cells also increase the expression of metastasis-related proteins (143), which may enable the acquisition of metastatic phenotypes by driving EMT (132).

Once disease presents and treatment commences, polyploid, and hyperdiploid cells remain key players driving the ongoing evolution of the patient disease. Elevated ploidy provides cells with multiple therapy-resistance mechanisms including infrequent cell cycle (110, 132), acquisition of primitive, therapy-resistant cell phenotypes (132, 158), over-expression of therapeutic targets leading to resistance (147), alternate pathway activation leading to therapy escape (144), as well as facilitating acquisition of the dreaded multi-drug resistant phenotype (148, 149). Polyploid tumor cells are created by cytotoxic and targeted therapies (149, 158–160), therefore the frequency of tumor cells with elevated ploidy is likely to significantly increase during therapy. Further, many front-line therapies are genotoxic mutagens. In this scenario, the therapy itself imparts a mutator phenotype onto the tumor, with polyploidy functioning as a genetic buffer to reduce the effects of deleterious mutations, increasing the probability of beneficial mutations surviving within the polyploidy sub-population to drive disease recurrence.

The adaptive capacity inherent to polyploidy cells means that even a small sub-population of surviving polyploid tumor cells are able to drive disease recurrence (39, 161). The capacity of polyploid tumor cells to repopulate post-therapy is likely to be significantly enhanced due to the ploidy-driven acquisition of a primitive cell phenotype with an elevated tumor-initiating capacity (132, 141), combined with a greatly reduced competition for resources due to the competing non-resistant tumor cells being killed off during therapy (162).

For these reasons, we predict that polyploid tumor cells play an integral role in disease recurrence and the acquisition of a therapy-resistant, increasingly malignant disease in patients during therapy.

## New Therapeutic Strategies that Target Polyploidy and Hyperdiploid Tumor Cell Subpopulations

Experiments in yeast and cancer model systems have shown that the presence of polyploidy generates points of fragility within cellular systems that can be targeted using specific therapeutics (163–168). These studies provide the critical proof-of-principle that polyploidy is in fact a druggable phenotype. However, the strategies proposed in these pioneering studies target stress responses or mitotic machinery, which poses the risk of increasing polyploidy in surviving cells (148, 149). Fortunately, recent studies have identified new avenues for therapy development that could be used in conjunction with established therapies to inhibit the formation of polyploidy tumor cells and decrease the adaptive capacity of tumors *in vivo*.

### Targeting metabolism to attack polyploidy and hyperdiploid tumor cells

Cell size scales linearly with DNA content in Eukaryotes (169–172). Cancer polyploid cells are proportionally larger than the euploid bulk population (110, 147), with giant polyploid cells being much larger than the euploid population (124). One potential consequence of large genome size and increased cell volume is a heightened metabolic demand, as bigger cells require more energy to grow to a sufficient cell volume to allow for cell doubling (117, 118). Further, the increased mRNA and protein expression caused by increased ploidy also demands more energy consumption (37). Consistent with increased genome size cell volume, and elevated transcription and translation, we noted polyploid tumor cells displayed a higher metabolic rate than the euploid control population (110).

The large cell size and increased metabolism of polyploid tumor cells may represent a point of fragility specific to the polyploid sub-population that could be exploited therapeutically. To test this hypothesis, we treated parental euploid and polyploid clonal cultures with the 2-deoxy-d-glucose, an established inhibitor of glycolysis (173–176). We found that the brain tumor polyploid cells were significantly more sensitive to the effects of glycolysis inhibition then the euploid parent control (110).

Critically, this increased dependence of polyploidy cells on glycolysis has been reported across several types of cancer. In acute myeloid leukemia (AML), Liu et al. demonstrated that targeting aurora kinases with specific inhibitors increased the prevalence of polyploidy in AML cells, and that AML polyploidy cells displayed increased glycolysis as measured by increased glucose uptake and lactic acid production (157). AML polyploidy cells were sensitive to the effects of 2-DG, suggesting that targeting metabolism may preferentially kill polyploidy tumor cells (157). mTOR is a conserved serine/threonine kinase that links cell signal transduction with cell metabolism and growth (177). Specific mTOR inhibitors promoted apoptosis and autophagy in polyploidy tumor cells and increased the efficacy of Aurora kinase inhibitors, confirming tumor metabolism as a viable point of therapeutic intervention against AML polyploidy tumor cells (157). Using breast cancer cells, Sharma et al. used the tyrosine kinase inhibitor BMS-777607 to induce polyploidization in breast cancer lines, and confirmed that therapy-induced polyploidy cells were resistant to the effects of a variety of chemotherapies (148). They then performed drug screens looking for additional inhibitors that specifically targeted polyploidy tumor cells, and identified that an inhibition of mTOR signaling prevented the formation of therapy-induced polyploidy and maintained the sensitivity of breast cancer cells toward the effects of chemotherapies (148). These results indicate that reducing polyploidy tumor cell formation by targeting metabolism may delay the evolution of therapy resistance (148). More recently, the same group revealed that BMS-777607 induced polyploidization in pancreatic cancer cells, which displayed pan-resistance to a range of chemotherapeutic compounds (160). Targeting metabolism using mTOR inhibitors reduced the formation of therapy-resistant polyploidy cells and synergized with BMS-770607, showing that for pancreatic tumor cells targeting tumor metabolism prevents the emergence of therapy-resistant polyploidy tumor cells (160).

Together, these results support the hypothesis that in brain, breast, leukemia, and pancreatic cancers, polyploid tumor cells have a commensurately higher metabolic requirement than euploid tumor cells, and that inhibiting metabolism is an effective therapeutic strategy to specifically target polyploid tumor cells to maintain tumors in a drug sensitive state.

### Stimulating AMP kinase activity using resveratrol and aspirin

Lissa et al. used an elegant high-throughput screening to screen a compound library for drugs that preferentially kill tetraploid cells, identifying resveratrol as an anti-tetraploid therapeutic agent (178). Resveratrol is an anti-fungal agent naturally occurring in grapes that has been reported to reduce tumor formation in a genetic mouse model of intestinal carcinoma when administered orally (179). Resveratrol stimulates AMPK activation by inhibiting phosphodiesterase 4 (PDE-4), allowing cAMP accumulation in cells and subsequent activation of protein kinase A (PKA) (180). Consistent with this being the primary mode of tetraploid killing, resveratrol treatment activated AMPK in tetraploid cells (180). Activation of AMPK using a separate PDE-4 inhibitor or over-expression of AMPK selectively killed tetraploid cells, whereas PKA inhibitors specifically blocked resveratrol killing (178). Aspirin (acetyl salicylate) and it is more active derivative salicylate also activate AMPK, and consistent with other AMPK activators were shown to selectively kill tetraploid cells (178). Using the mouse intestinal carcinoma cancer model, the Authors then confirmed that oral administration of either resveratrol or aspirin reduced the frequency of tetraploid intestinal epithelial cells (178), confirming that resveratrol and aspirin preferentially kill tetraploid cells *in vivo* using clinically relevant therapeutic doses (178).

Together, these data show that activating AMPK using the natural products resveratrol and aspirin can be used to specifically target tetraploid tumor cells *in vitro* and *in vivo*, potentially explaining the cancer-preventing effects of these two compounds reported using mouse models of tumor initiation (181). These findings support the exciting hypothesis that targeting tetraploid malignant precursor cells may become an effective chemopreventative strategy for humans.

## Conclusion and Future Directions

Cancer evolution has been intensely studied in recent years using cutting edge genomic approaches (182–185), and novel therapeutic strategies aimed at delaying tumor evolution are being developed using increasingly sophisticated and predictive computational models of tumor evolution (162, 186–189). Here, we have presented a complementary approach, which consists of identifying and therapeutically targeting the polyploid tumor cell subpopulations that are likely to facilitate rapid evolution. We have based our argument on evidence derived from yeast and cancer model systems, as well as from primary patient tumor samples, all of which support the hypothesis that polyploidy facilitates rapid evolution and the acquisition of therapy-resistant phenotypes in cancer patients.

We have also presented recent studies that identify promising new anti-polyploid therapeutic approaches, which could potentially be used to target polyploid tumor cells in cancer patients. We predict that the therapies stemming from these pioneering studies will be successfully translated and incorporated into novel anti-evolution therapies designed using systems biology approaches, which will significantly increase cancer patient lifespan by slowing the emergence of therapy resistance, as well as being used as chemopreventative agents to reduce the incidence of cancer.

## Conflict of Interest Statement

The authors declare that the research was conducted in the absence of any commercial or financial relationships that could be construed as a potential conflict of interest.
